# Different research designs and their characteristics in intensive care

**DOI:** 10.5935/0103-507X.20160050

**Published:** 2016

**Authors:** Wagner Luis Nedel, Fernando da Silveira

**Affiliations:** 1 Intensive Care Unit, Hospital Nossa Senhora da Conceição - Porto Alegre (RS), Brazil.; 2 Intensive Care Unit, Hospital de Clínicas de Porto Alegre - Porto Alegre (RS), Brazil.

**Keywords:** Research designs, Randomized clinical trial, Systematic review, Meta-analysis, Cohort studies

## Abstract

Different research designs have various advantages and limitations inherent to their main characteristics. Knowledge of the proper use of each design is of great importance to understanding the applicability of research findings to clinical epidemiology.

In intensive care, a hierarchical classification of designs can often be misleading if the characteristics of the design in this context are not understood. One must therefore be alert to common problems in randomized clinical trials and systematic reviews/meta-analyses that address clinical issues related to the care of the critically ill patient.

## Abstract (pt)

Os diferentes delineamentos de pesquisa apresentam diversas vantagens e limitações, inerentes às suas características principais. O conhecimento sobre o emprego adequado de cada um deles é de grande importância na aplicabilidade da epidemiologia clínica.

Em terapia intensiva, uma classificação hierárquica dos delineamentos, sem compreender suas peculiaridades neste contexto, pode muitas vezes ser errônea, devendo-se atentar para problemas corriqueiros em ensaios clínicos randomizados e em revisões sistemáticas/metanálises, que abordem questões clínicas referentes a cuidados de pacientes gravemente enfermos.

## INTRODUCTION

Epidemiology contributes to the development of different research methods that aim to answer clinical questions. Adequate knowledge of research designs is critical when planning research and when reading and interpreting studies, for which we recommend recent reviews on the subject.^(1-3)^

Our objective is to provide basic tools for identifying different designs, taking into account the peculiarities inherent to the intensive care context.

## EXPERIMENTAL STUDIES

Experimental studies are characterized by the artificial manipulation of an intervention by the researcher. In such studies, an intervention is administered, and its effect on the outcome is observed. Experimental studies are divided into the following categories:

**Randomized clinical trial (RCT)** - an interventional and prospective study. Participants should have the same opportunity to receive or not receive the proposed intervention. The groups should be as similar as possible so that the only difference between them is the intervention itself; the similarity of the groups enables the researcher to evaluate the effect of the intervention on outcomes in one group in relation to the other. The RCT is the gold standard for studies that aim to evaluate the effect of an intervention on the course of a clinical situation. It allows various biases to be eliminated because the intervention and control groups are allocated using random techniques and characteristics are distributed in a similar manner among both groups.^(3,4)^

The eligibility criteria for such a study can be numerous when the study needs to be reduced to a specific situation or can be simplified when the study needs to reflect clinical practice more closely. The criteria are designed to increase homogeneity among patients and strengthen the internal validity of the study. Treatment groups can be compared with one or more control groups in parallel, when there is parallel monitoring of more than one group, or crossed, when subjects are randomized to intervention and control groups and when the randomization sequence is reversed after clinical outcomes are measured. In such cases, each group receives both the intervention and control treatment, but at different times.

When the participants allocated to intervention and control groups are unaware of which treatment they are receiving, they are defined as "blinded" to the intervention type. Similarly, the researcher who administers, monitors and evaluates the intervention may not know which intervention the patient is receiving. When both patient and researcher are blinded to the intervention, the RCT is double-blind. Sometimes, especially in the intensive care environment, researchers or assistant staff cannot be blinded; in such cases, the researcher who evaluates the clinical outcome should be "masked" to the participant's allocation group (single-blind study).

**Nonrandomized clinical trial (quasi-experimental)** - In this type of study, there is an intervention group and a control group, but unlike in an RCT, patients are not allocated to each group randomly; instead, they are allocated according to the researcher's convenience.^(5)^ Controls can be contemporaneous (patients who are treated at the same time) or historical (e.g., information is obtained from medical records). Before-and-after studies are a form of nonrandomized trial. This design cannot control other factors that may have occurred simultaneously to the intervention deployed and that may have contributed to the change in outcome.^(5,6)^

## PREVALENCE STUDIES (CROSS-SECTIONAL)

Prevalence studies involve the simultaneous measurement of risk factors and outcomes. They cannot infer which came first, the exposure or the outcome.

## CASE SERIES

Case series report a given outcome in a group of patients, without the presence of a comparison group. They are useful for generating hypotheses to be tested in future studies.

## CASE-CONTROL STUDIES

Case-control studies are observational, longitudinal and retrospective. A population with a particular outcome of interest is selected (cases) along with another population that is similar to the first group but without the outcome of interest (controls). The two groups are compared, and the factors that may be related to the occurrence of the researched outcome are evaluated.

## COHORT STUDIES

Cohort studies are observational, longitudinal, prospective or retrospective. Populations that have and have not been exposed to the determined factor are selected and monitored for a specific period of time, after which the effect of the exposure factor on the occurrence of the outcome must be analyzed.^(7)^ This design has several purposes, such as evaluating risk factors for a particular disease, measuring the effects of prognostic factors or investigating diagnostic and therapeutic interventions.

## SYSTEMATIC REVIEW WITH META-ANALYSIS

In such studies, the object of analysis is not the patient, but research that has already been conducted on a particular topic. Original articles published in the literature are reviewed and selected systematically, and their results may be summarized under a single effect-size parameter (meta-analysis).^(1,8)^ Ideally, all the existing evidence on a given subject should be gathered, and more than one database should be used to search for articles.

## WHICH RESEARCH DESIGN IS THE BEST IN INTENSIVE CARE?

**Figure 1:**
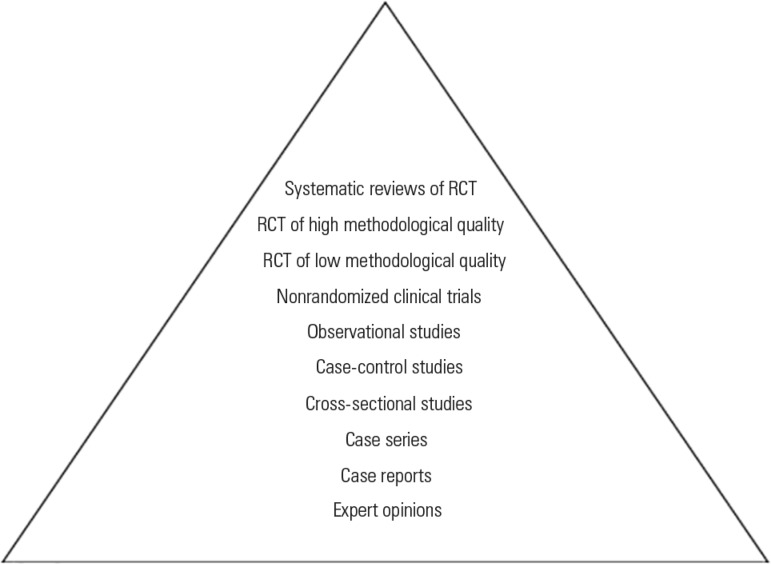
Traditional hierarchical classification of research designs. RCT - randomized clinical trial.

The RCT is defined as the "cornerstone" of clinical research according to the evidence-based medicine (EBM) spectrum. The hierarchical classification of designs, based on EBM principles, places RCT and meta-analyses derived from RCT at the tip of the pyramid, indicating that they represent the best possible methodological quality for answering a clinical question (Figure 1) because RCT are potentially less susceptible to bias than observational studies.^(9)^ However, in the context of intensive care, this hierarchy is often questionable, and it has even been suggested that in this scenario, RCT should be abandoned.^(10)^ It is important to understand that different designs have their advantages and limitations and that the study design used basically depends on the research question to be answered (Table 1).^(9)^

**Table 1:** Characteristics of the main research designs

Study design	Characteristics/applicability	Disadvantages
Randomized clinical trial	- Considered the gold standard for analyzing therapeutic interventions - The study groups differ only in terms of the intervention factor: there is direct inference of causality - Ideally, it is able to control selection bias and confounders that can affect the study outcome	- Expensive and time consuming - Not always feasible for ethical reasons - The absence of blinding, especially when it cannot be applied, may directly affect the study result - Subject to loss of patients during follow-up - Generally evaluates specific disease scenarios - Frequently conducted in academic scenarios, limiting the generalizability of the data
Nonrandomized clinical trial	- Performed when an RCT would be ideal but is not possible (due to cost or unacceptability to patients or managers) - Generally more practical than RCT - Used when attempting to demonstrate the efficacy of a new treatment (phase IV studies)	- Sample group is determined by convenience - Causality is not directly inferred - The effect of confounders may not be obvious
Before-and-after study	- Quick and easy to conduct - May be used to compare the ICU data or variables of a specific patient before and after an intervention is instituted - Useful in interventions that are difficult to perform using a clinical trial (e.g., to evaluate the efficacy of isolation measures in the transmission of nosocomial infections by multiresistant germs)	- Subject to the Hawthorne effect - Simultaneous interventions may alter the studied outcome and the interval between the measurement of outcomes - Subject to the “regression toward the mean” phenomenon
Cross-sectional study	- Measures the prevalence of a particular outcome - One of the first stages in investigating the cause of disease outbreaks - May be used to analyze frequencies of risk factors and study outcomes - Low cost and easy to conduct	- Measures of exposure and disease are performed at the same time, which reduces the study’s ability to establish a causal association (association does not necessarily imply causation) - Difficult to use for investigating low-prevalence conditions - Appropriate when evaluating diagnostic tests or disease outbreaks
Case series	- The characteristics of the study population are related - Useful when characterizing rare disease scenarios with a diagnosis or treatment that is not clearly established in the literature (for example: the characterization of outbreaks of rare micro-organisms)	- There is no comparison group (reference) - Sampling is not representative of the studied population - Causal associations cannot be evaluated
Case-control	- Cheaper and faster (than cohort studies) - Useful when investigating disease causes, especially when the incidence of the outcome (disease) is rare or has a long latency period, as the starting point is the outcome and a retrograde analysis of risk factors is performed	- There is no way of knowing the incidence of the disease - Prone to sampling bias among both cases and controls and to observation and recording bias
Cohort	- Provides the best information about the etiology, incidence and natural history of a disease by starting with risk factors and analyzing subsequent outcomes - Useful when risk factors have a low prevalence - A cohort study should be used when it is not possible to conduct an RCT for ethical reasons	- Expensive and requires long periods of observation - Subject to loss to follow-up - Unable to control all confounders - Subject to discontinuity of patient follow-up - Frequently presents results that are discordant with those of RCT that analyze the same research question
Systematic review with meta-analysis	- Clinical research with the lowest evidence level - Summarizes the results of primary studies using strategies that decrease the occurrence of random and systematic errors	- The quality of a meta-analysis depends on the quality of the studies it includes - The greater the heterogeneity of the included studies, the lower the reliability of the result obtained

RCT - randomized clinical trial; ICU - intensive care unit.

RCT conducted in the intensive care unit (ICU) usually produce negative results; unrealistic therapeutic expectations often lead to inaccurate estimates for the sample size calculation and the outcome incidence at baseline.^(11)^ Inadequate records taken prior to the trial's execution and changes in the protocol or sample size during the course of the study are also frequent events. This raises the question of how events that occur during the course of a trial can affect the study design and, consequently, the reported results. The results of RCT also have limited generalizability because of high exclusion rates and the results obtained in the control group, which may differ from the real-life context in which the results must be applied. The management of critically ill patients entails their exposure to numerous physiological and therapeutic variables that can potentially mask the outcome of a given intervention; such changes may lead to greater losses in the interpretation of results.^(10)^

Even systematic reviews with meta-analysis have important limitations that prevent them from being fully applicable to clinical practice due to the inclusion of trials with low methodological quality and potential publication biases.^(8,12)^ Small studies tend to have a higher incidence of beneficial effects in the intervention group, which may be at least partly explained by the lower methodological quality of such studies.^(13)^ This phenomenon has been demonstrated in meta-analyses in critical care setting.^(14)^

Over time, cohort studies have improved the quality of information available for determining a course of action, especially with regard to comparative efficacy research. Such studies can see beyond the clinical trial, particularly because they include longer patient follow-up, larger study populations and better analysis of uncommon outcomes.^(15)^ Observational studies are an important complement to RCT; as a general rule, they obtain answers more efficiently and have greater generalizability.^(16)^ There are various statistical tools available to minimize the effects of confounding biases in observational studies, such as group pairing, sample stratification, multivariate analysis and propensity scores, which should be used whenever feasible.
